# Identification and expression of small multidrug resistance transporters in early‐branching anaerobic fungi

**DOI:** 10.1002/pro.4730

**Published:** 2023-09-01

**Authors:** Susanna Seppälä, Taylor Gierke, Elizabeth E. Schauer, Jennifer L. Brown, Michelle A. O'Malley

**Affiliations:** ^1^ Department of Chemical Engineering University of California Santa Barbara Santa Barbara California USA; ^2^ Bioengineering Program University of California Santa Barbara California USA; ^3^ Joint BioEnergy Institute (JBEI) Emeryville California USA

**Keywords:** anaerobic fungi, bioprocessing, membrane proteins, small multidrug resistance transporters, yeast

## Abstract

Membrane‐embedded transporters impart essential functions to cells as they mediate sensing and the uptake and extrusion of nutrients, waste products, and effector molecules. Promiscuous multidrug exporters are implicated in resistance to drugs and antibiotics and are highly relevant for microbial engineers who seek to enhance the tolerance of cell factory strains to hydrophobic bioproducts. Here, we report on the identification of small multidrug resistance (SMR) transporters in early‐branching anaerobic fungi (Neocallimastigomycetes). The SMR class of transporters is commonly found in bacteria but has not previously been reported in eukaryotes. In this study, we show that SMR transporters from anaerobic fungi can be produced heterologously in the model yeast *Saccharomyces cerevisiae*, demonstrating the potential of these proteins as targets for further characterization. The discovery of these novel anaerobic fungal SMR transporters offers a promising path forward to enhance bioproduction from engineered microbial strains.

**Abbreviations**
GFP: green fluorescent protein
PDB: protein data bank
SMR: small multidrug resistance

## 1 INTRODUCTION

Microbial production of value‐added chemicals from renewable feedstocks is a sustainable alternative to petroleum‐based production streams. For microbial cell factories to become economically viable, it is necessary to acquire efficient substrate conversion and uninhibited product formation. Key bottlenecks are the intracellular accumulation of often toxic products and insufficient substrate uptake and conversion (Boyarskiy & Tullman‐Ercek, 2015; van der Hoek & Borodina, 2020). The flux and yields of microbial cell factories can be modulated by modifying the repertoire of membrane‐embedded transporters (Malla et al., 2022; Podolsky et al., 2021; Seppälä et al., 2019; Wang et al., 2021; Zhang et al., 2020). Engineering microbial strains encoding efflux transporters have enhanced the tolerance to bioprocessing conditions, including solvents and potentially toxic products (Chen et al., 2013; Dunlop et al., 2011; Jiménez‐Bonilla et al., 2020). Attractive targets for metabolic engineering are drug transporters that can extrude a wide variety of molecules, such as transporters belonging to the primary active ATP‐binding cassette (ABC) superfamily of transporters; or the secondary active major facilitator superfamily (MFS) (Chen et al., 2013; Dunlop et al., 2011; Ma et al., 2021).

One transporter family that is underexplored for bio‐based production applications is the small multidrug resistance (SMR) family (Bay et al., 2008). SMR transporters are abundant in the bacterial kingdom but have not previously been reported in eukaryotes. In bacteria, SMR transporters reside in the plasma membrane, where they couple the extrusion of structurally diverse substrates, such as quaternary ammonium compounds, to the influx of protons (Bay et al., 2008). These proteins are often implicated in antibiotic resistance and also serve as promising engineering targets to increase the tolerance of microbial production strains to hydrophobic bioproducts, biofuels, and ionic liquids (Higgins et al., 2019; Kermani et al., 2018; Saleh et al., 2018; Slipski et al., 2020). In spite of their biotechnological potential, SMRs have not been explored in the context of microbial bioproduction. One reason may be that membrane proteins as such remain somewhat overlooked in the microbial engineering and bioproduction fields, which historically have been focused more on biosynthetic pathways catalyzed by soluble enzymes. Also, SMRs are typically considered a prokaryotic protein family and have not been tested as a tool to enhance the performance of eukaryotic bioproduction organisms such as yeast.

Here, we have identified genes encoding putative SMR transporters in early diverging anaerobic fungi, which reside in the guts and rumen of large herbivores. The anaerobic fungi (Neocallimastigomycetes) are known for the synthesis and secretion of carbohydrate‐active enzymes (CAZymes) (Gruninger et al., 2018; Solomon et al., 2016; Theodorou et al., 2005), but have recently been shown to also have useful sugar transport systems (Podolsky et al., 2021) and membrane proteins that confer solute tolerance (Seppälä et al., 2019). An investigation of publicly available genomes from prokaryotes and eukaryotes suggests that Neocallimastigomycetes are the only sequenced eukaryotes that harbor genes encoding SMR‐like proteins (Clark et al., 2016; Grigoriev et al., 2014; Sayers et al., 2021; The UniProt Consortium, 2023). While the exact function of the transcribed genes remains unknown, the finding offers exciting possibilities to deploy these underused proteins in microbial production hosts such as *Saccharomyces cerevisiae*.

## 2 RESULTS AND DISCUSSION

### 2.1 Fungal SMR‐like proteins are unique to anaerobic fungi

**FIGURE 1:**
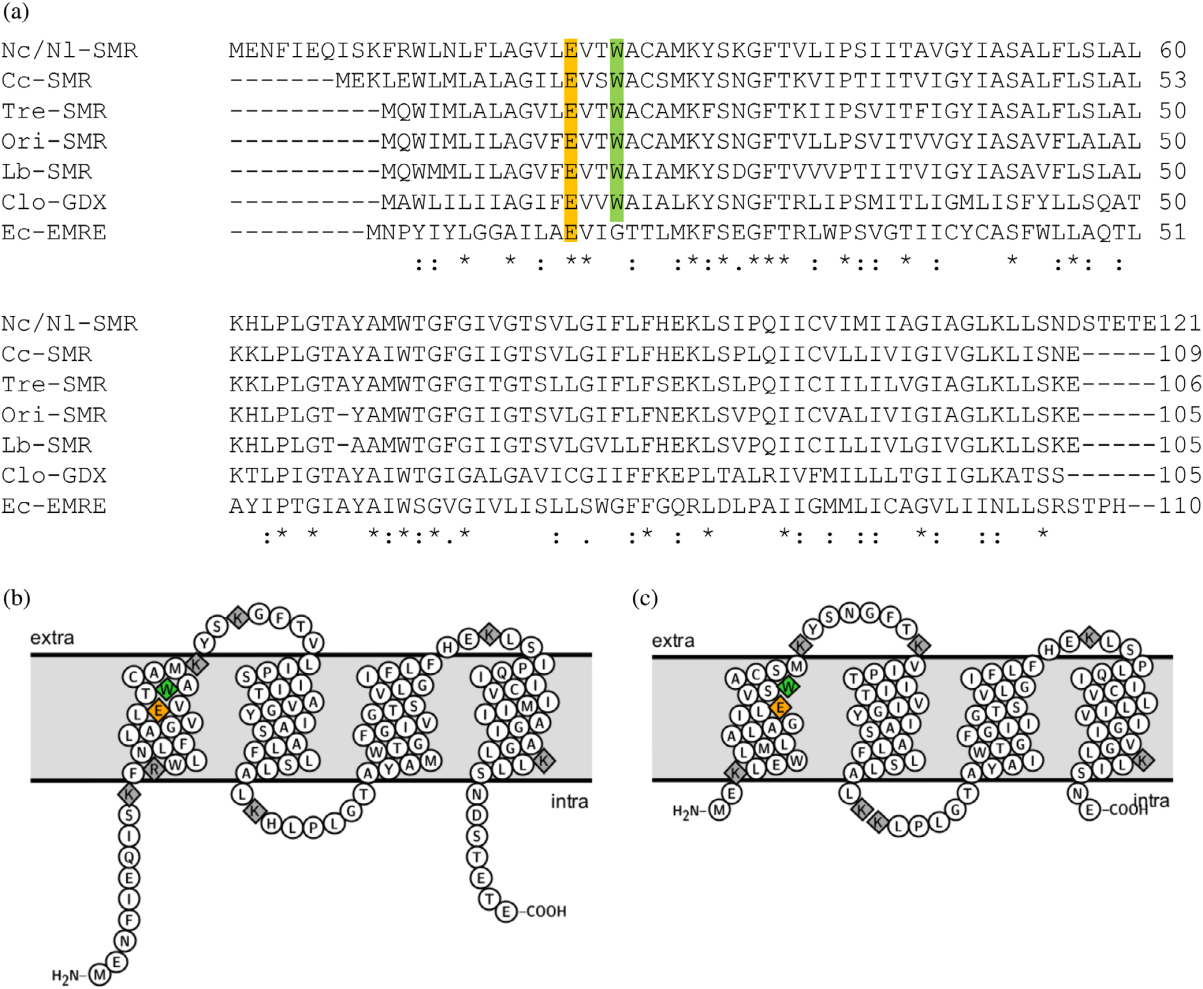
Anaerobic fungi encode putative SMR transporters that are homologous to bacterial SMRs. (a) A Clustal Omega alignment of SMR amino acid sequences from the identical *N. californiae* and *N. lanati* sequences Nc/Nl‐SMR, *C. churrovis* Cc‐SMR, as well as the three top NCBI BLAST hits: SMR sequences from *Treponema* sp. Tre‐SMR (GenBank: MBQ1628041.1), *Oribacterium* sp. Ori‐SMR (GenBank: MBO5598130.1), and *Lachnospiraceae* sp. Lb‐SMR (GenBank: MBR1853192.1). The sequences of functionally characterized *Clostridium* sp. Clo‐GDX (GenBank: 6WK8_A) and *Escherichia coli* Ec‐EMRE (GenBank: Z11877.1) are also included. (b,c) Membrane protein topology diagrams based on TOPCONS predictions of the topology of *N. californiae/lanati* SMR (left) and *C. churrovis* SMR (right), made using the Protter visualization tool. The conserved glutamate and tryptophan residues in transmembrane helix 1 are highlighted in orange and green in all panels. The positively charged amino acid residues K and R are highlighted in gray in panels b and c.

SMR transporters belong to the Interpro Small drug/metabolite transporter protein family (IPR000390). A search for IPR000390 proteins in published fungal genomes on the Mycocosm database, which comprises hundreds of genomes from across the fungal tree of life, revealed that this protein family is only present in the early diverging fungal phylum Neocallimastigomycetes, specifically the isolated and characterized strains *Neocallimastix californiae*, *Neocallimastix lanati*, and *Caecomyes churrovis*. Each of these three strains appears to have a single gene encoding a putative SMR transporter: the coding sequences of the *N. californiae* and *N. lanati* genes appear identical, whereas the *C. churrovis* sequence is 71% identical to the *N. californiae* and *N. lanati* sequences (Figure 1a and Data S1). The GC‐content of the genes is 30%–34% GC, and none of the genes appear to have introns.

A BLAST search of GenBank and Uniprot, using the gut fungal sequences as query, returns only hits from Neocallimastigomycetes and prokaryotes (Data S2; Clark et al., 2016; Sayers et al., 2021; The UniProt Consortium, 2023; Altschul et al., 1990). This result supports the general consensus that while SMR transporters are ubiquitous in prokaryotes, they appear to be absent in eukaryotes that have been sequenced to date (Bay et al., 2008; Du et al., 2015). Given the high degree of horizontal gene transfer previously reported in anaerobic fungal genomes (Haitjema et al., 2017; Murphy et al., 2019; Wang et al., 2019), it is possible that the Neocallimastigomycetes have acquired SMR proteins from prokaryotes in the rumen microbiome to enable their adaptation and ability to compete with other organisms.

Transcriptomic data collected from *N. californiae*, *N. lanati*, and *C. churrovis* corroborated that the gut fungal genes are transcribed in many different culture conditions (Brown, Swift, Mondo, Seppala, Salamov, Singan, Henrissat, Drula, et al., 2021; Solomon et al., 2016; Wilken et al., 2021). Published transcriptional data for these three fungal strains reveals that these genes are actively transcribed in a monoculture cultivation condition, but some putative SMR genes are expressed at low levels (e.g., lower transcripts per million counts) when grown on a range of substrates such as glucose, avicel, alfalfa stem, and corn stover.

### 2.2 Fungal SMRs are highly similar to homologous proteins found in rumen bacteria

Given that the SMR‐encoding gene sequences are identical, the predicted protein sequences from *N. californiae* (ID 696754) and *N. lanati* (ID 1618129) are also identical, and 75% identical to the SMR protein sequence from *C. churrovis* (ID 21219). NCBI BLAST reveals that the top protein hits, sharing 79%–80% identity with the *N. californiae/N. lanati* SMR, are sequences found in bacterial *Oribacterium* sp., *Lachnospiraceae* sp., and *Treponema* sp., which are commonly found in ruminant microbiomes (O'Donnell et al., 2017; Peng et al., 2021; Xie et al., 2021) (Figure 1a and Data S2). As highlighted in Figure 1, the negatively charged glutamate that is present in position 23 in *N. californiae/N. lanati* SMR and position 16 in the *C. churrovis* SMR is conserved in SMRs and involved in the binding of both substrate and protons (Li et al., 2021). Further, the tryptophan found at position 26 in the *N. californiae/N. lanati* SMR and position 19 in the *C. churrovis* SMR is conserved across guanidinium exporters, whereas other SMR proteins, such as the well‐characterized *Escherichia coli* EmrE, contain a conserved glycine or alanine in the corresponding position (Figure 1a) (Kermani et al., 2020). When aligned to a diverse sample of SMRs, the *N. californiae/N. lanati* and *C. churrovis* SMRs and their homologs from ruminal bacteria cluster near bacterial guanidinium exporters (Figure S1).

Both the *N. californiae/N. lanati* SMR sequence and the *C. churrovis* SMR sequence are predicted to have four transmembrane segments, which is the canonical motif for bacterial SMR proteins (Figure 1b,c) (Bay et al., 2008; Omasits et al., 2014; Tsirigos et al., 2015). Intriguingly, the gut fungal SMR‐like proteins identified in this study exhibit a charge distribution that is very similar to so‐called dual‐topology SMRs. While typical bacterial SMR transporters form dimers composed of two protomers that are predicted to adopt opposite orientations in the membrane (Fleishman et al., 2006; Kermani et al., 2020; Lloris‐Garcerá et al., 2012), other SMRs are known to form homodimers made of two oppositely orientated but otherwise identical polypeptide chains (Bay et al., 2008; Rapp et al., 2006). These proteins are characterized by weak topological signals, among which the most important is the number and distribution of positively charged amino acid residues lysine and arginine (Seppälä et al., 2010; von Heijne, 1989). As is seen in Figure 1b,c, both fungal proteins have very few positively charged amino acid residues that are predicted to be evenly distributed on both sides of the membrane (Seppälä et al., 2010). De facto dual‐topology membrane proteins are rare in nature and appear to be virtually absent in eukaryotic proteomes, but these findings suggest that anaerobic fungi may be hosts to transporters with unusual architectures although further experiments are required to determine the topology of the proteins in the eukaryotic membranes (von Heijne, 2006). A 3D structure prediction using ColabFold suggests that the *N. californiae/lanati* SMR folds into a bundle of four alpha‐helices that can be almost flawlessly superimposed on top of Clo‐GDX (PDB ID: 6WK5), and Ec‐EMRE (PDB ID: 7MH6, 7JK8, 7SFQ; Kermani et al., 2020; Kermani et al., 2022; Mirdita et al., 2022; Pettersen et al., 2004; Shcherbakov et al., 2021; Shcherbakov et al., 2022; Figure S2).

### 2.3 Gut fungal putative SMR transporters can be expressed in *S. cerevisiae* membranes

**FIGURE 2:**
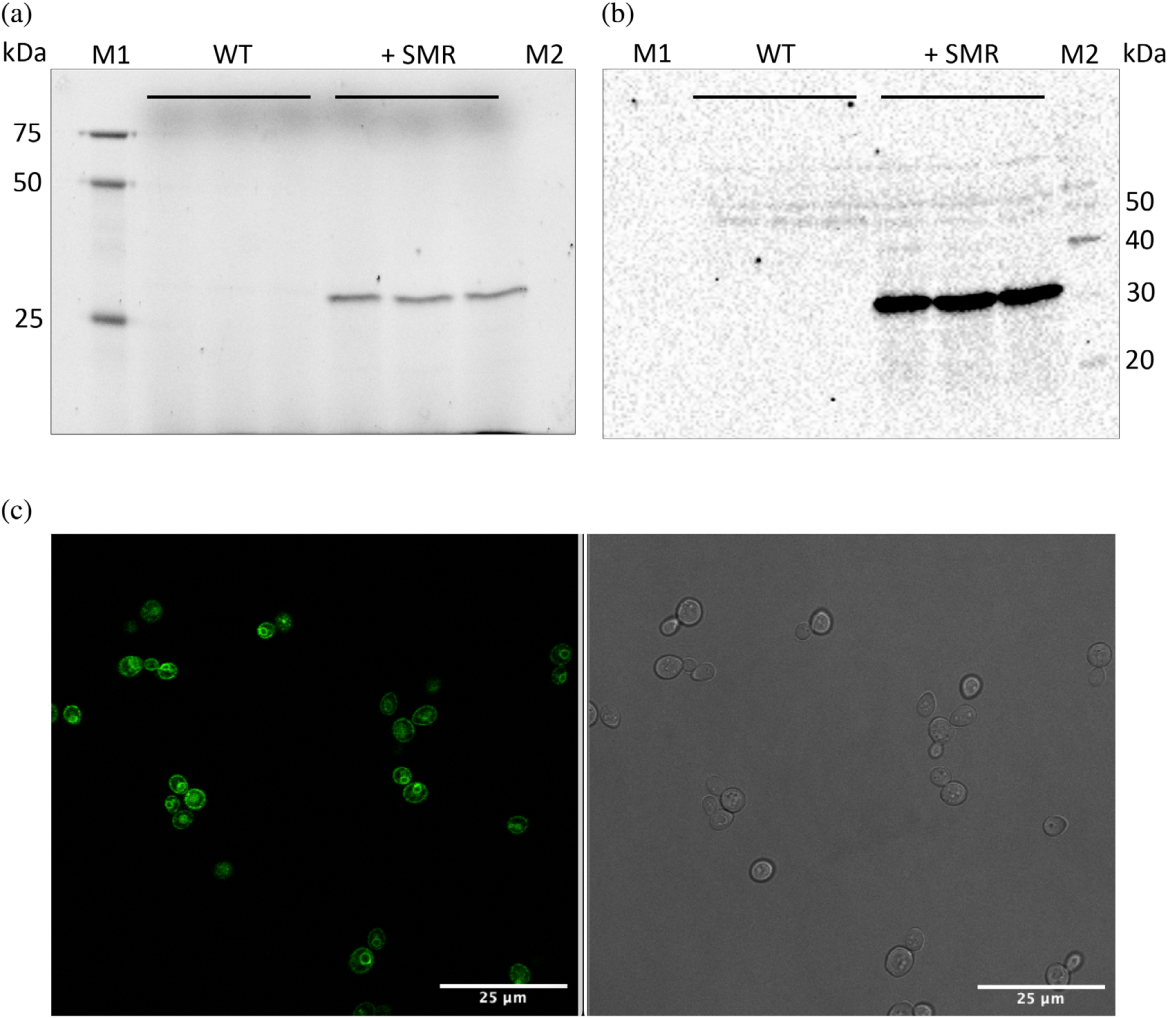
Fungal SMRs are produced and membrane‐localized in yeast. (a) In‐gel fluorescence of wild‐type *S. cerevisiae* BJ5465 (WT) and *S. cerevisiae* BJ5465 harboring *N. californiae* SMR‐GFP‐His10 (+SMR) in triplicate. M1: Precision Plus Protein WesternC Standard. M2: MagicMark XP Western Protein Standard. (b) Western blot using an anti‐GFP antibody. Samples are identical to the ones shown in panel a. (c) Confocal microscopy shows localization of *N. californiae* SMR‐GFP‐His10 in yeast cells. GFP fluorescence (left) and transmitted light (right). The scale bar is 25 μm.

To enable downstream functional characterization of newly identified fungal SMR proteins, we cloned a codon‐optimized version of *N. californiae SMR* for expression in the model yeast *S. cerevisiae* (Data S3). The gene was cloned such that the resulting protein was equipped with a carboxyterminal Green Fluorescent Protein (GFP) moiety. As is shown in Figure 2, the resulting protein was produced without any apparent signs of degradation or aggregation, as judged from the in‐gel fluorescence of the GFP moiety and the single band in the Western Blot using a polyclonal anti‐GFP antibody (Figure 2a,b; Geertsma et al., 2008; Newstead et al., 2007). Confocal microscopy revealed that the protein appears to be targeted to the endoplasmic reticulum and trafficked to the surface of the yeast cell, indicative of proper membrane localization and folding. Ni‐NTA purification yielded ~0.5 mg protein from 1 L of yeast culture.

### 2.4 The function of fungal SMRs remains elusive

Bacterial SMR transporters are known to increase the resistance of microbial cells to a range of quaternary ammonium compounds and cationic dyes, with each protein class selective for different substrates (Bay et al., 2008). One major group of SMR transporters is the quaternary ammonium compound subtype, which confers resistance to compounds such as cetyltrimethylammonium bromide and ethidium bromide and includes the well characterized *E. coli* EmrE (Bay et al., 2008; Peng et al., 2021). Recently it has been suggested that the majority of SMR proteins function as guanidinium exporters (Kermani et al., 2018). Given the sequence homology of our putative *N. californiae* SMR to guanidinium exporters, we aimed to functionally characterize our SMR in *S. cerevisiae* cells using growth‐based toxicity assays against guanidinium, ethidium bromide, and cetrimonium bromide. We were, however, unable to find any significant difference in growth between wild‐type *S. cerevisiae* cells or cells expressing the putative SMR and wild‐type *S. cerevisiae* cells in the tested conditions (Figure S3). One reason may be that the fungal protein is not fully functionally folded in the yeast. The fact that the GFP‐moiety, which is fused to the carboxyterminal end of the membrane protein, remains fluorescent and appears at the periphery of the cells indicates that the membrane protein is folded and inserted into the membrane (Drew et al., 2008). It is, however, possible that the fungal SMR protein requires an as yet unidentified protein partner or cofactor to perform its function. Finally, although the Nc‐SMR is similar to SMRs and in particular guanidinium exporters, it is possible that we have not yet identified the correct substrate or screening conditions in the heterologous organism.

## 3 MATERIALS AND METHODS

### 3.1 Identification of SMR genes

A search for the Interpro entry IPR000390 across all published fungal genomes deposited on JGI Mycocosm revealed that SMR sequences are only present in Neocallimastigomycete fungi (Blum et al., 2021). Subsequently, the sequences of *N. californiae/N. lanati* SMR and *C. churrovis* SMR were used as a bait to blast all the genomes that were deposited on JGI Mycocosm on July 21, 2022 (Grigoriev et al., 2014). Genes encoding putative SMR proteins were also identified in differential transcriptomic data collected from anaerobic gut fungi (Brown, Swift, Mondo, Seppala, Salamov, Singan, Henrissat, Henske, et al., 2021; Seppälä et al., 2016; Solomon et al., 2016; Wilken et al., 2021). NCBI BLAST was done using default parameters (Altschul et al., 1990). Clustal Omega was used for sequence alignments (McWilliam et al., 2013). Transmembrane segments were predicted using TOPCONS (Tsirigos et al., 2015). Membrane protein topology diagrams were drawn using the Protter visualization tool (Omasits et al., 2014).

### 3.2 Cloning and expression of SMR genes in yeast

A gene encoding *N. californiae* SMR was codon optimized for expression in yeast and flanked by recognition sites for restriction enzymes EagI and SacII (Genewiz, South Plainfield, NJ). The gene was subcloned into the integrating pITy vector (Parekh et al., 1996). In the pITy vector, the gene of interest is downstream of a GAL1 promoter and fused at the 3′ end to a Green fluorescent protein‐His10 tag (O'Malley et al., 2009). The pITy‐NcSMR‐GFP‐His_10_ plasmid was transformed into *S. cerevisiae* BJ5465 (Mat**a** *ura3‐52 trp1 leu2∆1 his∆200 pep4::HIS3 prb∆1.6R can1*; ATCC no. 208289) using the lithium acetate/PEG method (Daniel Gietz & Woods, 2002). Cells were selected on YPD broth (10 g/L yeast extract; Becton, Dickinson & Co, Sparks, MD), 20 g/L peptone extract (Becton, Dickinson & Co, Sparks, MD), 20 g/L dextrose (Sigma‐Aldrich, St. Louis, MO) supplemented with 15 g/L agar (Becton, Dickinson & Co, Sparks, MD) and 500 μg/mL Geneticin (Sigma‐Aldrich, St. Louis, MO). Expression was performed as described in Seppälä et al. (2019), with few modifications. Briefly, single colonies were inoculated into YPD broth and cultured at +30°C, 225 rpm, for ~16 h. These cultures were subsequently used to seed YPR broth (10 g/L yeast extract (Becton, Dickinson & Co, Sparks, MD), 20 g/L peptone extract (Becton, Dickinson & Co, Sparks, MD), 20 g/L raffinose (Thermo Fisher Scientific, Waltham, MA) at a starting OD_600_ of 0.12–0.2, where OD_600_ is the optical density of the culture at 600 nm. Following cultivation at +30°C, 225 rpm to an OD_600_ of 0.6–1, NcSMR‐GFP‐His_10_ expression was induced by addition of galactose at a final concentration of 2% w/v. Induced cultures were harvested for downstream assays after 16–20 h.

### 3.3 SDS‐PAGE, in‐gel fluorescence, and Western blot

SDS‐PAGE and in‐gel fluorescence were performed as described in (Drew et al., 2008), with few modifications. Briefly, 5 ODU of yeast cultures were collected by centrifugation, resuspended in 50 μL Yeast Protein Extraction Reagent (Thermo Fisher Scientific, Waltham, MA), and incubated at room temperature for 30 min. Exactly 1 ODU equals the amount of cells that yields OD_600_ = 1 in 1 mL of liquid. The samples were mixed with 50 μL Laemmli sample buffer ×2 at 37°C for 30–60 min. A total of 0.5 ODU of each sample was loaded on a 12% Tris‐Glycine gel together with Precision Plus Protein WesternC Standard (Bio‐Rad, Hercules, CA) and MagicMark XP Western Protein Standard (Thermo Fisher Scientific, Waltham, MA). A GFP antibody (Abcam no. ab6663) was used for Western blotting using Pierce ECL Substrate (Thermo Fisher Scientific, Waltham, MA) according to manufacturers' instructions. In‐gel GFP fluorescence and Western blot bands were visualized using ChemiDoc MP Imaging system (Bio‐Rad, Hercules, CA).

### 3.4 Confocal microscopy

Exactly 2 ODU of yeast cultures were collected using centrifugation and washed with ×1 PBS. Cells were added to glass microscopy slides coated with 0.1% w/v poly‐l‐lysine (Sigma‐Aldrich, St. Louis, MO). Confocal micrographs were acquired using a Leica SP8 Resonant Scanning Confocal microscope equipped with a ×60 objective, and fluorescence was imaged using a GFP filter.

### 3.5 Purification of NcSMR‐GFP‐His_10_ from yeast membranes

One liter of cultures of yeast containing NcSMR‐GFP‐His_10_ were induced as described above. After ~20 h induction, cells were harvested by centrifugation and washed with 10 mM Tris–HCl, pH 8. For lysis, cell pellets were mixed with one volume 0.5 mm zirconium beads in cell resuspension buffer (50 mM Tris–HCl pH 7.6, 1 mM EDTA, 0.6 M sorbitol, Pierce protease inhibitor cocktail; Thermo Fisher Scientific, Waltham, MA). Cells were lysed by 6 cycles of vigorous vortexing for 2 min and chilling on ice for 2 min. The lysate was cleared by centrifugation at ~4000*g* and membranes were collected by ultracentrifugation at ~230,000*g* for 60 min. Using a Potter‐Elvehjem homogenizer, membranes were resuspended in 20 mM Tris–HCl pH 7.5, 0.3 M sucrose, 0.1 mM CaCl_2_ at a final concentration of 1 g membrane/mL. Proteins were solubilized with 2% n‐Dodecyl‐β‐D‐Maltopyranoside (Anatrace, Maumee, OH) in 20 mM Tris–HCl pH 8, 10 mM imidazole, Pierce protease inhibitor cocktail and agitated at +4°C for 1 h. The solubilized proteins were mixed with 1:1 Ni‐NTA resin and batch purified using manufacturer's instructions (Thermo Fisher Scientific, Waltham, MA). Briefly, Ni‐NTA with bound protein was repeatedly washed by centrifugation at 700*g* until the supernatant OD_280_ ~ 0, each time using two resin‐bed volumes of wash buffer (20 mM Tris–HCl, 300 mM NaCl, pH 8, 0.1% n‐Dodecyl‐β‐d‐Maltopyranoside [Anatrace, Maumee, OH], 25 mM imidazole). The protein was eluted by centrifugation at 700*g* using 20 mM Tris–HCl pH 8, 300 mM NaCl, 0.1% n‐Dodecyl‐β‐d‐Maltopyranoside (Anatrace, Maumee, OH), 250 mM imidazole. Imidazole was removed using PD10 desalting columns (VWR, Radnor, PA), and the final protein concentration was determined using the Pierce BCA Protein Assay Kit (Thermo Fisher Scientific, Waltham, MA).

### 3.6 Toxicity assays

Exactly 3 mL cultures containing NcSMR‐GFP‐His_10_ were induced as described above. Induced cultures were used to seed 200 μL cultures in YPG broth (10 g/L yeast extract, Becton, Dickinson & Co, Sparks, MD), 20 g/L peptone extract (Becton, Dickinson & Co, Sparks, MD), 20 g/L galactose (Thermo Fisher Scientific, Waltham, MA) in a Corning polystyrene clear‐bottom 96‐well plate (Thermo Fisher Scientific, Waltham, MA) at a starting OD_600_ of 0.1. The cultures were supplemented with either guanidinium hydrochloride (0.625–2.25 mM), ≤60 μM ethidium bromide or ≤10 μM cetrimonium bromide and OD_600_ of the cultures was measured every 15 min for a duration of 24 h using a Tecan Infinite M1000 microplate reader (Männedorf, Switzerland).

### 3.7 Structural 3D prediction

The 3D structure prediction was done using ColabFold v1.5.2 with the Nc‐SMR protein sequence as the query sequence (Drew et al., 2008). Protein structures were visualized with UCSF Chimera (Pettersen et al., 2004). Based on structural similarity, the rank 2 model of the Nc‐SMR was superimposed onto the best‐aligning chains of Clo‐GDX (PDB ID: 6WK5) and Ec‐EMRE (PDB ID: 7MH6, 7JK8, 7SFQ) using the Chimera MatchMaker command (Kermani et al., 2020, 2022; Shcherbakov et al., 2021, 2022).

## 4 CONCLUSIONS

Here, we identified SMR‐like proteins in three strains of anaerobic gut fungi, which are known for their remarkable ability to break down recalcitrant biomass. To the best of our knowledge, this is the first report of such proteins in any eukaryotic organism. We have demonstrated that these proteins can be produced in and purified from membranes of the model *S. cerevisiae*. Although the function of the fungal proteins remains elusive, acquisition of the SMRs may contribute to the resistance of anaerobic fungi to antimicrobial agents in the gut microbiome. These proteins are a promising novel target for future characterization and microbial engineering efforts that seek to enhance the tolerance of microbial strains for bioprocessing applications.

## CONFLICT OF INTEREST STATEMENT

The authors declare no conflicts of interest.

## Supporting information


**Data S1.** Anaerobic fungal nucleotide and protein sequences.

**Data S2.** Top 1000 prokaryotic BLAST hits using the *N. californiae/lanati* SMR protein sequence as query.

**Data S3.** DNA sequence of codon‐optimized *N. californiae smr‐gfp‐his10*.

**Figure S1.** Phylogeny of a subset of SMR family proteins.

**Figure S2.** Predicted 3D structure of *N. californiae/lanati* SMR superimposed on SMRs of known structure.

**Figure S3.** Growth of yeast cultures with and without a gene encoding *N. californiae smr‐gfp* in the presence of putative SMR substrates.
