# Supplementary material for: Identification and expression of small multidrug resistance transporters in early‐branching anaerobic fungi
Source: Protein Sci. 2023 Sep 1;32(9):e4730. doi: 10.1002/pro.4730 (PMC10443351; doi:10.1002/pro.4730)
Supplement: Supplementary file 1 — Data S1. Anaerobic fungal nucleotide and protein sequences. [file PRO-32-e4730-s002.docx]

**Supplementary information 1: Anaerobic fungal nucleotide and protein sequences**

**Transcript sequences**

Sequences of *Neocallimastix californiae* G1 v. 1.0 (Neosp1|696754), *Neocallimastix lanati* v. 1.0 (Neolan1|1618129), and *Caecomyces churrovis* A v1.0 (Caecom1|21219) are available at the Joint Genome Institute Mycocosm portal, <https://mycocosm.jgi.doe.gov/mycocosm/home>.

BLUE: untranslated regions or noncoding exon sequences

RED: coding regions

***Neocallimastix californiae* G1 v. 1.0 (Neosp1|696754) transcript**

AAAATTTGTTTTTGTTATTATGCATATAATTATTTTTATTATAATTTAATATTATAAATACAAACACTCCTATTCTTTTTTTATATTTTTATATATATTATAAAATTAAAAATGGAAAATTTTATTGAACAAATAAGCAAGTTTCGTTGGCTAAATTTATTCTTAGCAGGAGTACTTGAGGTTACTTGGGCTTGTGCCATGAAATATTCAAAAGGTTTTACTGTTCTTATTCCTTCTATTATAACTGCTGTGGGTTACATTGCAAGTGCCTTATTTCTTTCTCTAGCACTTAAACATTTACCACTTGGAACCGCTTATGCCATGTGGACAGGTTTCGGAATTGTAGGAACTTCTGTCCTTGGTATTTTCTTGTTTCATGAGAAATTATCAATTCCTCAAATTATATGTGTTATTATGATTATTGCTGGTATTGCTGGACTAAAATTATTATCCAATGATTCAACGGAAACAGAATAAAAGTAATAATAATTCATAACTTTGAACGAATTTATATATTCATTTACTTAATTTTAAAAAATAAAAGATAT

***Neocallimastix lanati* v. 1.0 (Neolan1|1618129) transcript**

AAAAAAAAAAAAAAATTTGTTTTTGTTATTATGCATATAATTATTTTTATTATAATTTAATATTATAAATACAAACACTCCTATTCTTTTTTTATATTTTTATATATATTATAAAATTAAAAATGGAAAATTTTATTGAACAAATAAGCAAGTTTCGTTGGCTAAATTTATTCTTAGCAGGAGTACTTGAGGTTACTTGGGCTTGTGCCATGAAATATTCAAAAGGTTTTACTGTTCTTATTCCTTCTATTATAACTGCTGTGGGTTACATTGCAAGTGCCTTATTTCTTTCTCTAGCACTTAAACATTTACCACTTGGAACCGCTTATGCCATGTGGACAGGTTTCGGAATTGTAGGAACTTCTGTCCTTGGTATTTTCTTGTTTCATGAGAAATTATCAATTCCTCAAATTATATGTGTTATTATGATTATTGCTGGTATTGCTGGACTAAAATTATTATCCAATGATTCAACGGAAACAGAATAAAAGTAATAATAATTCATAACTTTGAACGAATTTATATATTCATTTACTTAATTTTAAAAAATAAAAGATATATTTATAAATGAT

***Caecomyces churrovis* A v1.0 (Caecom1|21219) transcript**

Transcript(823 bp)/CDS Sequence(330 bp)

AAATAATATTTAACATTTAATAATACTTAATAATGAACAATGCTAAACAATTATTGGATAAAAAAAATACATTAATAATTCCATAAATGAATATTTGGTTATTAAAAAAAAAATTTTTTTTTTCTGATAAAATATTTCTTATTATTGAAGGTAGATAAAAAAAAAAAAAAAAAAAAAAAAAAATGGAAAAATTGGAATGGCTAATGCTTGCACTTGCAGGTATTCTGGAAGTATCTTGGGCTTGTTCAATGAAATATTCAAACGGTTTTACAAAAGTAATTCCGACCATAATAACCGTTATTGGTTATATTGCAAGTGCATTGTTTTTGTCCCTTGCTCTGAAAAAGTTGCCGCTTGGCACGGCATATGCAATATGGACAGGATTCGGTATTATTGGAACTTCTGTTCTTGGAATTTTTCTTTTTCATGAAAAATTATCACCTCTACAAATCATTTGTGTTTTACTGATCGTAATCGGTATTGTCGGATTAAAATTAATTTCAAATGAGTAAAAAAAAAAAAAAAAAAAAAAAAATAAATATGTATTAAAAATAAATGCTTATATAATTAAAACAGACTAAACCACATCAACTAATATTTATTTATAACAAGTTGTAAATGTATAATAAATTACTCTTTAAAAAAAAAAGATTTATCAAATACATTTTAATTATTTATAAAATAATTATAATTACAGTTACAGTAAAGCACATTTAAAATATGAATTAATATAAAATGTATAACAGAAATATAATAATATATATACATTATTATTTAAGTACATTGTAGTAACTATGGTATAAATAAATTTATAGTATTAATA

**Protein sequences**

Translations made assuming that the Neocallimastigomycete fungi adhere to the universally conserved codon table.

***Neocallimastix californiae* G1 v. 1.0 (Neosp1|696754) translated sequence**

MENFIEQISKFRWLNLFLAGVLEVTWACAMKYSKGFTVLIPSIITAVGYIASALFLSLALKHLPLGTAYAMWTGFGIVGTSVLGIFLFHEKLSIPQIICVIMIIAGIAGLKLLSNDSTETE*

***Neocallimastix lanati* v. 1.0 (Neolan1|1618129) translated sequence**

MENFIEQISKFRWLNLFLAGVLEVTWACAMKYSKGFTVLIPSIITAVGYIASALFLSLALKHLPLGTAYAMWTGFGIVGTSVLGIFLFHEKLSIPQIICVIMIIAGIAGLKLLSNDSTETE*

***Caecomyces churrovis* A v1.0 (Caecom1|21219) translated sequence**

MEKLEWLMLALAGILEVSWACSMKYSNGFTKVIPTIITVIGYIASALFLSLALKKLPLGTAYAIWTGFGIIGTSVLGIFLFHEKLSPLQIICVLLIVIGIVGLKLISNE*
