# Supplementary material for: Identification and expression of small multidrug resistance transporters in early‐branching anaerobic fungi
Source: Protein Sci. 2023 Sep 1;32(9):e4730. doi: 10.1002/pro.4730 (PMC10443351; doi:10.1002/pro.4730)
Supplement: Supplementary file 3 — Data S3. DNA sequence of codon‐optimized N. californiae smr‐gfp‐his10. [file PRO-32-e4730-s001.docx]

**Supplementary information 3: DNA sequence of codon optimized *Neocallimastix californiae* *smr-gfp***

The SacII restriction enzyme site (ccgcgg) between the *N. californiae* *smr* and the start of *gfp* is in lowercase bold font.

5’-ATGGAAAATTTTATCGAACAGATCAGCAAGTTTCGCTGGCTGAACCTGTTTCTGGCCGGTGTGCTGGAAGTGACCTGGGCCTGCGCCATGAAGTATAGCAAGGGCTTCACCGTGCTGATCCCGAGCATTATTACCGCCGTGGGCTATATTGCCAGCGCCCTGTTTCTGAGCCTGGCCCTGAAACACCTGCCGCTGGGCACTGCCTATGCCATGTGGACCGGTTTCGGTATTGTGGGCACTAGCGTGCTGGGTATCTTCCTGTTCCATGAAAAGCTGAGCATCCCGCAGATCATCTGCGTTATTATGATTATCGCCGGCATTGCCGGTCTGAAACTGCTGAGCAATGACAGCACCGAAACCGAA**ccgcgg**ATGGTGAGCAAGGGCGAGGAGCTGTTCACCGGGGTGGTGCCCATCCTGGTCGAGCTGGACGGCGACGTAAACGGCCACAAGTTCAGCGTGTCCGGCGAGGGCGAGGGCGATGCCACCTACGGCAAGCTGACCCTGAAGTTCATCTGCACCACCGGCAAGCTGCCCGTGCCCTGGCCCACCCTCGTGACCACCCTGACCTACGGCGTGCAGTGCTTCAGCCGCTACCCCGACCACATGAAGCAGCACGACTTCTTCAAGTCCGCCATGCCCGAAGGCTACGTCCAGGAGCGCACCATCTTCTTCAAGGACGACGGCAACTACAAGACCCGCGCCGAGGTGAAGTTCGAGGGCGACACCCTGGTGAACCGCATCGAGCTGAAGGGCATCGACTTCAAGGAGGACGGCAACATCCTGGGGCACAAGCTGGAGTACAACTACAACAGCCACAACGTCTATATCATGGCCGACAAGCAGAAGAACGGCATCAAGGTGAACTTCAAGATCCGCCACAACATCGAGGACGGCAGCGTGCAGCTCGCCGACCACTACCAGCAGAACACCCCCATCGGCGACGGCCCCGTGCTGCTGCCCGACAACCACTACCTGAGCACCCAGTCCGCCCTGAGCAAAGACCCCAACGAGAAGCGCGATCACATGGTCCTGCTGGAGTTCGTGACCGCCGCCGGGATCACTCTCGGCATGGACGAGCTGTACAAGCACGTGTCATCACATCATCATCATCATCATCATCATCATCATTAA-3’
