# Supplementary figures and images for: Identification and expression of small multidrug resistance transporters in early‐branching anaerobic fungi
Source: Protein Sci. 2023 Sep 1;32(9):e4730. doi: 10.1002/pro.4730 (PMC10443351; doi:10.1002/pro.4730)

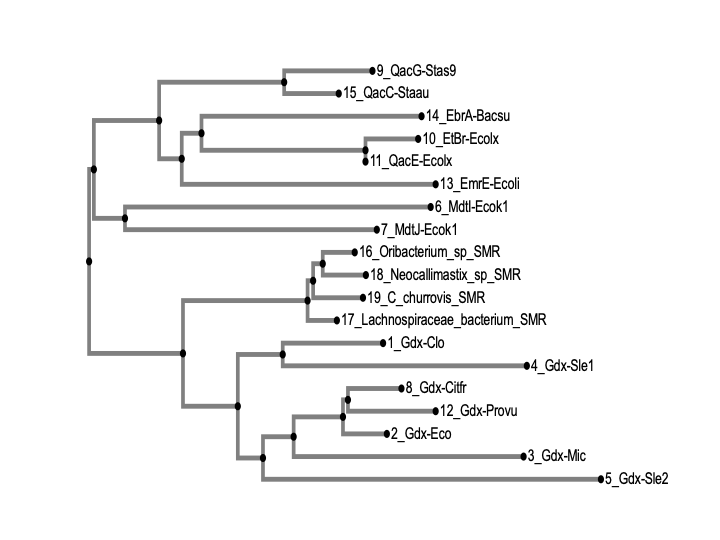

Supplement: Supplementary file 4 — Figure S1. Phylogeny of a subset of SMR family proteins. [file PRO-32-e4730-s006.tiff]

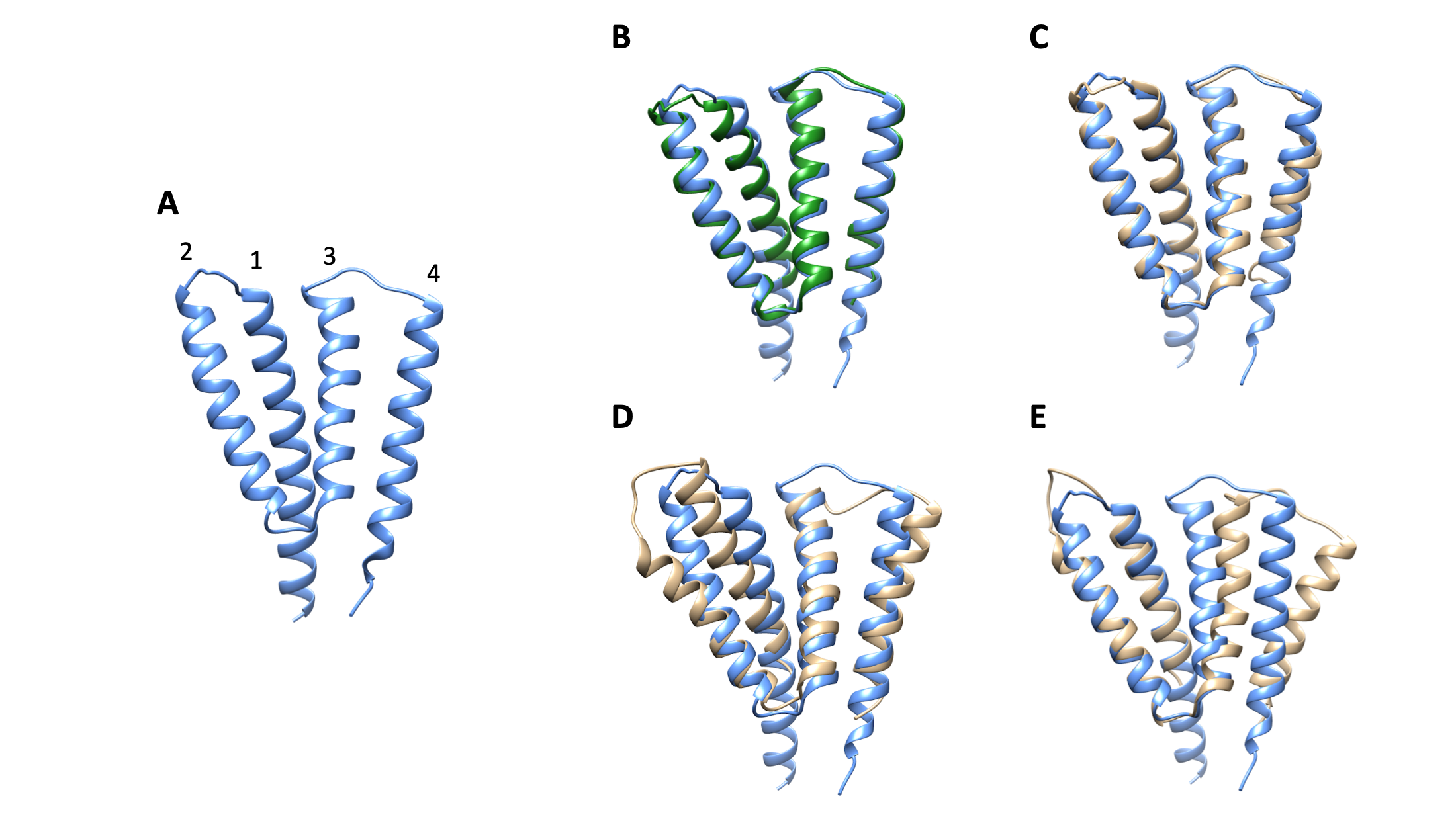

Supplement: Supplementary file 5 — Figure S2. Predicted 3D structure of N. californiae/lanati SMR superimposed on SMRs of known structure. [file PRO-32-e4730-s004.tiff]

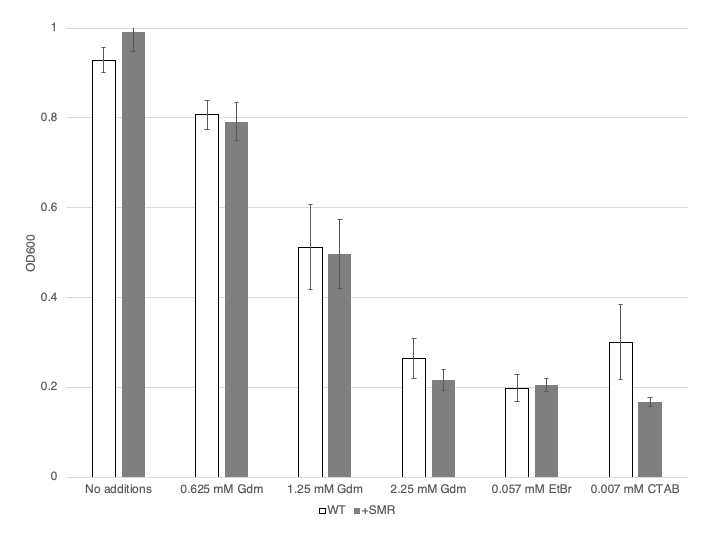

Supplement: Supplementary file 6 — Figure S3. Growth of yeast cultures with and without a gene encoding N. californiae smr‐gfp in the presence of putative SMR substrates. [file PRO-32-e4730-s005.tiff]
